# Assessment of three alternative methods for bacterial disinfection of hatching eggs in comparison with conventional approach in commercial broiler hatcheries

**DOI:** 10.1371/journal.pone.0283699

**Published:** 2023-03-30

**Authors:** Gerzon Motola, Hafez Mohamed Hafez, Sarah Brüggemann-Schwarze

**Affiliations:** Department of Veterinary Medicine, Institute of Poultry Diseases, Freie Universität Berlin, Berlin, Germany; Foshan University, CHINA

## Abstract

The disinfection of commercial hatching eggs before incubation is a common strategy to reduce potential vertical transmission of bacterial and fungal infections from the eggshell to one-day-old chicks that may prevail in poultry products and eventually reach the end consumer. The present investigation focuses on the parallel testing and application of four different disinfection methods (conventional and alternative) under commercial hatchery conditions against natural eggshell bacterial contamination. Hatching eggs from two ROSS 308 broiler breeder flocks were selected and divided into six different groups: two groups were not disinfected and served as negative controls, and four were independently disinfected following product specifications and protocols. From each group, a sample of 100 hatching eggs was selected for bacterial re-isolation, utilizing a modified shell rinse method. Colony-forming units (cfu) from the shell rinse suspensions were determined and analyzed to establish cfu values for each tested egg. These values were analyzed to determine the bacterial disinfection capacity of the four disinfection methods under commercial hatchery conditions. The tested methods were hydrogen peroxide + alcohol, peracetic acid, low energy electron beam, and the gold standard in practice: formaldehyde. Among these methods, formaldehyde, peracetic acid, and low energy electron beam showed a significant difference when compared to the non-disinfected groups whereas hydrogen peroxide + alcohol did not. The bacterial disinfection capacity of the tested methods was compared as well to the gold standard method formaldehyde fumigation and only low energy electron beam achieved similar disinfection levels as formaldehyde. According to our data, three methods significantly reduce the bacterial load on the eggshell of hatching eggs under commercial hatching conditions, including potential alternative methods such as low energy electron beam that perform similar to the gold standard in practice.

## Introduction

Poultry meat has become a high-quality protein source for consumers worldwide. During the last decades, poultry meat production has become increasingly efficient [1–3]. To maintain this highly efficient production, several aspects should be considered, such as nutrition [4, 5], housing and rearing [6, 7] and health [8]. Health and hygiene management practices have been established to avoid the spread of infections throughout the entire production chain [8, 9].

The broiler production pyramid starts with the hatching eggs laid by the grand and parent flocks. Generally, the hatching eggs are disinfected in the hatchery previous incubation to avoid the introduction of external pathogens. This step has proven to be effective to prevent vertical infection of one-day-old chicks that may otherwise prevail in poultry products and eventually reach the end consumer [10].

The eggshell presents a mixed bacteria population [11] that originates from the cloaca of the laying hen [12, 13] as well as the contact with the laying nest [14, 15]. The surface of the eggshell is formed by an intricate mineral mesh with pores that permits oxygen and humidity exchange [16–18], but also allow bacteria to penetrate the eggshell and infect the eggs interior [19, 20]. Once the egg is laid, a change of temperature occurs that generates a vacuum of the egg’s internal structure, allowing the surface bacteria to enter through the pores [21].

Hatching egg disinfection represents a big challenge for the industry due to the fact that a disinfection is required that not only works on the surface of the egg but ideally also penetrates the pores without affecting the viability of the embryo, hatchability or general performance of the hatched chicks. In a previous trial, six disinfection methods were tested to assess their disinfection efficacy against an artificial contamination model with an Extended-spectrum beta-lactamase (ESBL) producing *Escherichia coli* (*E*. *coli*) strain [22]. In a following experiment, disinfection was performed on artificially contaminated broiler hatching eggs, to assess the disinfection efficacy and also the impact of the disinfection methods on hatchability, mortality and bodyweight [23].

The present investigation focuses on testing different disinfection methods (conventional approaches and alternative ones) against the eggshell bacterial contamination under commercial hatchery conditions. Neither the parent flocks nor the used hatching eggs in the present investigation were artificially contaminated.

Previous studies have already compared the disinfection efficacy as well as the adverse effects of various disinfection methods (formaldehyde, hydrogen peroxide + alcohol, peracetic acid and low energy electron beam) under *in-vitro* conditions [22–26] demonstrating the effectiveness of the disinfectants against various types of bacteria but they have never been compared on a large scale or under field conditions. By performing parallel field trials, we considered the practicability of the methods, providing us a better idea of the true performance and suitability of each disinfection method for the industry. Results were compared to analyze whether novel methods could serve as an alternative method for bacterial disinfection to the gold standard in practice: formaldehyde. Hatching egg disinfection with formaldehyde has been proven to have a broad-spectrum antibacterial efficacy [27–29], however, due to growing concerns regarding potential carcinogenic, mutagenic and toxic side effects [28, 30], novel methods are being evaluated as alternatives for producers to use under field conditions. In this study we aim to identify alternative methods to formaldehyde that can offer efficacious and practicable bacterial disinfection at commercial hatchery level.

## Material and methods

### Egg delivery, storage, and division of the disinfection groups

Hatching eggs from two broiler breeder flocks (Nr. 424 and Nr. 426), from the line ROSS 308 were selected for the trials. Both flocks were at the 14^th^-16^th^ production week and located on the same breeder farm in Germany.

From each flock, eggs were collected from the farms using regular hatchery transport. Due to the size of the flocks and to complete the required number of hatching eggs for each trial, eggs were stored at 18°C, 70% humidity, for a maximum of 2 days; no disinfection was performed prior arrival to the hatchery to avoid any disinfection bias. An optical screening was performed to avoid batches with an excess of dirt. Eggs were then stored on trolleys containing 36 trays with 126 eggs each.

The disinfection methods tested in this study are formaldehyde, hydrogen peroxide + alcohol, peracetic acid and low energy electron beam. A graphical representation of the experimental design can be found in S1 Fig.

Four groups (A -D) were established using eggs from both flocks (half of the amount from each). Each group consisted of 27,216 hatching eggs. Apart from Group A, non-disinfected control group, each group was treated with one disinfection method: Group B) formaldehyde (reference method), Group C) hydrogen peroxide + alcohol, Group D) peracetic acid in micro cages.

As soon as the required number of eggs was achieved for each group, they were disinfected accordingly and kept in storage at 18°C and 75% humidity. All disinfection methods were performed following the parameters and protocols described in a previous publication [22], with application protocols adapted to the higher amount of hatching eggs as shown in Table 1.

**Table 1:** Disinfection methods and protocols used during trials. Application protocols were performed according to the information provided by the producer.

Commercial name	Active substance	Application method	Concentration	Application protocol
**Jäklechemie® Formaldehyd Biozid 20%**	Formaldehyde	Fumigation	44 ml/m^3^	15 min fumigation + 10 min neutralization with ammoniac + 5 hours ventilation
**Wessoclean® K50 Goldline**	Hydrogen peroxide + ethanol	Fine Spray	Ready-to-use product	1 min spraying + 50 min exposure time in chamber
**Kesla ® 1+1 Wofasteril SC super**	Peracetic acid in micro-cages	Foaming	1% = 1 ml peracetic acid + 1 ml foaming agent	Foaming of the eggs with pressure foamier + 1 drying time before incubation
**Evonta® Ebeam Prototype**	Low energy electron beam	Radiation	200 keV, 60 kGy	1 sec exposure time for each side of the egg (2 sides radiated)*

*The prototype of the Low energy electron beam is equipped with a mechanism to turn over the eggs without having any external contact to avoid potential contamination and to ensure a 360° disinfection.

In the case of the low energy electron beam group (Group E) an independent trial was performed due to complex logistics that the disinfection machine required for the trials. A total of 2,268 hatching eggs were selected exclusively from one flock (Nr. 426) and disinfected using a prototype machine, in which single eggs were positioned on the disinfection chamber and disinfected. As a comparison group, 2,268 eggs from the same flock (Nr. 426) were disinfected with formaldehyde (as in Group B) and labelled as a formaldehyde Group B^1^.

### Sampling of eggs

For groups A-D, 100 eggs were tested after disinfection: 50 from each flock. In group E and B^1^, 100 eggs exclusively from one flock (Nr. 426) were used for the trials. For all groups, eggs were sampled from similar locations on trolleys and trays. From all six trolleys conforming each group the two that were positioned in the middle of the group during disinfection were selected for sampling, the middle tray pulled out and the eggs located in the middle of the tray chosen. Using this sampling technique, we assured to pick eggs from all groups eliminating any positioning bias.

All selected eggs were picked using sterile latex gloves, changing gloves between groups. Each group was sampled and transported separately to avoid any potential cross-contamination.

### Bacterial re-isolation using modified shell rinse method

For the re-isolation of bacteria, a modified version of the shell rinse method [31] was performed. Each egg was placed in sterile WHIRL-PAK® STAND UP BAGS, 18 oz. where 10 ml of sterile lysogeny broth (LB) was added. Bags were closed and placed on an orbital shaker at room temperature (between 21–26°C) for 20 min at 125 rpm (revolutions per minute). After shaking, broth was taken from each bag to determine the colony formation units (cfu). As an enrichment method to isolate extended-spectrum beta-lactamase (ESBL) producing bacteria, the bags were sealed again and incubated at 37°C overnight (18–24 hrs.) and sampled afterwards.

### Determination of colony forming units (cfu)

Colony forming units of the shell rinse suspensions were determined using: 1) Plate count (PC) agar for total bacterial count, 2) MacConkey agar no. 3 (MacC) for *Enterobacteriaceae*, 3) Columbia Horse Blood CNA Agar (CNA) for Gram-positive cocci (*Staphylococci* and *Streptococci*), 4) Brilliance UTI Clarity™ Agar (UTI) for coliforms and *Enterococci*, 5) Brilliance MRSA 2 Agar (MRSA) for the screening of methicillin-resistant *Staphylococcus aureus*, *and 6)* MacConkey agar no. 3 + Cefotaxime 4μg/ml (ESBL) for the screening of ESBL and AmpC producing bacteria.

As plating method, a triple droplet method was used with a serial dilution, depending on the disinfection group [32]. From each dilution, triplicates of 10μl were dripped onto the solid medium surface. All suspension samples were dripped onto all five media and incubated for 18–24 hrs. at 37°C. As for the overnight culture, 100 μl from each sample was spread across the MacConkey + Cefotaxime media surface and incubated for 18–24 hrs. at 37°C.

After incubation, all media were analyzed to establish cfu values for each tested egg. Random colonies from the different media were picked and analyzed using Matrix-Assisted Laser Desorption Ionization Time of Flight Mass Spectrometry (MALDI-TOF) Microflex LT® and Biotyper database® (Bruker Daltonics, Germany).

### Data analysis

Data were analyzed using the statistical program SPSS. The normality distribution of the data was analyzed using the Shapiro-Wilk test. Since the data was not normally distributed, the one-way ANOVA (Kruskal-Wallis test) was used to assess the significance of the disinfection efficacy of each method compared to the non-disinfected group and to evaluate the difference between the groups. For the comparison between flocks, the Mann-Whitney U test was performed for each disinfection method. The statistical level of significance was set at 5 percent for all analyses (p ≤ 0.05). A significance value adjustment was made by the Bonferroni correction for multiple tests.

Data from Group E (low energy electron beam) were compared to the value of Group A (Non-disinfected) and B^1^ (Formaldehyde repetition) since they were subject of an independent trial.

### Ethics statement

After laying and transportation to the hatchery, the hatching eggs were stored at 18°C to prevent embryo development. The hatching eggs utilized for this trial were disinfected and tested before the egg incubation at the hatchery started.

According to the German Animal welfare legislation (Tierschutz-Versuchstierverordnung, 2013) [33] studies performed with chicken eggs do not require ethics approval when the study is accomplished before hatching and no suffering or pain is expected for the animals that continue to live after hatch (TierSchVersV § 14). In a previous study [23], it was demonstrated that the tested disinfection methods had no negative effect on the embryo development or hatchability.

The disinfected eggs that were not selected as samples for this trial underwent standard commercial incubation and hatching process after disinfection and were excluded from further participation in the study. Additional investigation into the hatchability and broiler performance were carried out in parallel studies by a separate research team [34, 35] and the findings were not included in the present investigation.

## Results

Bacteria were re-isolated from the surface of non-disinfected eggs (control group) and four groups of disinfected eggs. The eggs were collected from two different parent flocks (flock 424 and 426). Among the six different media that were used during the trial, only plate count medium (PC) showed growth along all repetitions of the control group (non-disinfected) and was selected for compassion of the disinfection efficacy during the data analysis. Randomly selected colonies that grew on PC, CNA, and UTI media were used for 16S rRNA genomic sequencing [36, 37] in order to determine the bacterial species that were resistant to the different disinfection methods. No bacterial growth occurred on the MRSA and ESBL media in any of the repetitions.

**Fig 1:**
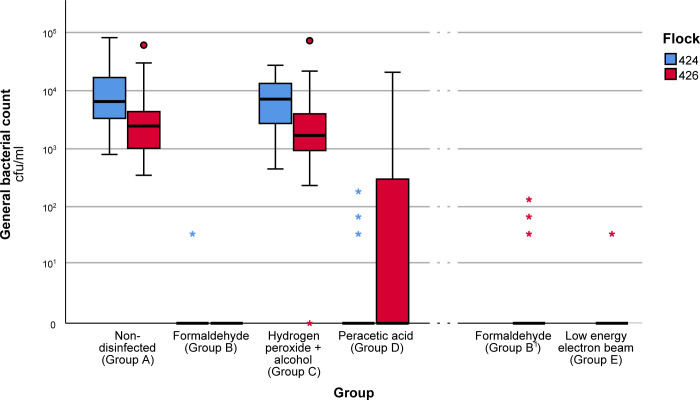
Re-isolation rates of bacteria found on the eggshell with eggshell rinse method from control and disinfection groups. The re-isolation rates are divided by group and flock. The number of eggs tested per group was 100 with n = 50 per flock. Formaldehyde Group B^1^ and low energy electron beam (Group E) present a total of 100 samples from only one flock. The box represents the interquartile range, the line the median of the samples, the circle represents a cluster of outlier values, while the asterisk represents a single outlier value. The data is representative for one of the two repetitions (for repetition see S2 Fig.).

The total bacteria count of each group is presented in Fig 1, showing the variation depending on the disinfection methods as well as the flock in which the trial was conducted. The mean re-isolation rate of the non-disinfected group (Group A) was 7.8 x 10^3^ cfu/ml with a standard error of 9.1 x 10^2^ and served as a control group.

When comparing the re-isolation rates of the tested methods, we observed that formaldehyde and low energy electron beam presented a low re-isolation rate of bacteria on the eggshell, when compared to the non-disinfected control group, with less than 5 of the 100 tested samples presenting bacterial growth after treatment per group. While the mean values of the formaldehyde and low energy electron beam group were 1.9 cfu/ml with a standard error of 14.4 cfu/ml and 0.1 cfu/ml with a standard error of 0.166 cfu/ml respectively, other disinfection methods did not perform as efficacious. In the hydrogen peroxide + alcohol group a mean 8.7 x 10^3^ cfu/ml with a standard error of 8.2 x 10^2^ cfu/ml was recorded, and in the peracetic acid with micro cages group a mean of 5.1 x 10^2^ cfu/ml with a standard error of 2.7 x 10^2^ cfu/ml was calculated.

There was a significant statistical difference between the two different flocks for each group, except for formaldehyde Group B, which was not statistically significant (p = 0.782). Formaldehyde Group B1 and low energy electron beam were only tested in flock 426.

When compared against the non-disinfected group (control group), all groups presented a statistical significance except for the group with hydrogen peroxide, as shown in Tables 2 and 3.

**Table 2:** Statistical data analysis of the different disinfection methods including non-disinfected as control group. The analysis was performed using the cfu/ml count of both flocks and two trials combined per group.

Group	Non-disinfected (Group A)	Formaldehyde (Group B)	Hydrogen peroxide + alcohol (Group C)	Peracetic acid in micro cages (Group D)
Non-disinfected (Group A)		<0.001^a^	**1.000** ^**b**^	<0.001^a^
Formaldehyde (Group B)	<0.001^a^		<0.001^a^	0.032^a^
Hydrogen peroxide + alcohol (Group C)	**1.000** ^**b**^	<0.001^a^		<0.001^a^
Peracetic acid in micro cages (Group D)	<0.001^a^	0.032^a^	<0.001^a^	

^a^ Significant difference between groups

^b^ Not a significant difference between groups

**Table 3:** Statistical data analysis of the low energy electron beam independent disinfection trial including non-disinfected as control group and formaldehyde as golden standard. The analysis was performed using the cfu/ml count of two trials combined per group and the historical non-disinfected re-isolation values of flock 426.

Group	Non-disinfected (Group A)	Formaldehyde (Group B^1^)	Low energy electron beam (Group E)
Non-disinfected (Group A)		<0.001^a^	<0.001^a^
Formaldehyde (Group B^1^)	<0.001^a^		**1.000** ^**b**^
Low energy electron beam (Group E)	<0.001^a^	**1.000** ^**b**^	

^a^ Significant difference between groups

^b^ Not a significant difference between groups

The groups that presented a significant difference with a p<0.001 when compared with the non-disinfection group were: formaldehyde, peracetic acid in micro cages and low energy electron beam. Hydrogen peroxide + alcohol did not show a significant difference to the non-disinfected group.

The disinfection efficacy of the tested methods was compared, using the re-isolation rate values, to the reference method: formaldehyde fumigation. Only low energy electron beam achieved similar disinfection efficacy as formaldehyde (p> 0.05) as shown in Table 3.

To examine, which bacterial species resisted the disinfection process, representative isolates were picked from colonies that had been re-isolated for each group and identified using MALDI-TOF and 16S rRNA genomic sequencing. The re-isolated bacteria mainly belonged to gram-positive bacteria, specifically the *Staphylococcus* species as shown in Table 4.

**Table 4:** Species identification of random isolates from control and disinfected groups using MALDI-TOF and 16S rRNA genomic sequencing.

Disinfection method / Group	Bacteria species re-isolated
Non-disinfected (Group A)	*Staphylococcus hyicus Staphylococcus lentus*
Formaldehyde (Group B)	*Bacillus mojavensis Bacillus subtilis*
Hydrogen peroxide + alcohol (Group C)	*Staphylococcus lentus Aerococcus viridans*
Peracetic acid in micro cages (Group D)	*Staphylococcus lentus Staphylococcus epidermidis Streptococcus alactolyticus*
Low energy electron beam (Group E)	*Staphylococcus epidermidis Lysinibacillus fusiformis*

The lack of growth in the MacC agar predicted a mostly gram-positive species identification with MALDI-TOF. The samples were selected for the diverse agars but mainly UTI and CNA. In the case of the re-isolation on the formaldehyde and low energy electron beam group, the colonies that presented growth on any of the plates were selected for comparison with other groups.

## Discussion

The disinfection of hatching eggs, is a practice that has been performed for a long time, with studies dating back to the beginning of 1900s showing the usage of disinfection methods in combination with artificial incubation [38]. There are several hatching egg disinfection methods in use worldwide [39–41] under field conditions that claim to have benefits on a deeper level, such as in the pores, without interfering with hatchability or viability of hatched chicks. In previous studies, an *in vitro* test was performed to analyze the bacterial disinfection efficacy [22], and potential adverse effects of various disinfection methods on hatchability and embryo viability were tested [23, 34, 35].

In the present investigation, we focused on the application of four previously tested methods under hatchery field conditions and compared the results to the gold standard formaldehyde fumigation.

The non-disinfected group presented an average re-isolation rate of 7.8 x 10^3^ cfu/ml, consisting of mainly gram-positive bacteria. Previous studies have shown the sensitivity of gram-negative bacteria to desiccation and temperature changes in comparison to gram-positive [42], which could be an explanation for the lack of re-isolation of enterobacteria. The eggs were collected every day and transported from the farm to the hatchery and remained untouched until the trial started (stored at room temperature for a maximum of 2 days). During this time, part of the bacteria could have desiccated, reducing the re-isolation capacity. The general re-isolation rate varied between both non-disinfected control flocks, ranging from 5.2 x 10^3^ in flock 426 to 1.4 x10^4^ in flock 424.

For low energy electron beam a prototype had to be used for the field trial. The eggs were placed by hand one-by-one into the machine that could disinfect up to 16 eggs at a time. The eggs were then disinfected from one side, turned over (with a mechanical system) and disinfected from the other side. Due to the limited number of eggs that could be disinfected at the time, as well as the fact that a prototype was used for the trial, the results of low energy electron beam (Group E) are not comparable to the groups B-D.

Formaldehyde treatment, which is considered the gold standard method, effectively disinfected all of the eggs in the trails. On only 4 out of 200 tested eggs showed re-isolation of bacteria after formaldehyde treatment, with an average re-isolation rate of 1.9 cfu/ml (with a standard error of 14.4 cfu/ml). This re-isolated colonies could have been present due to an incomplete disinfection or post-disinfection contamination. The bacteria re-isolated in the non-disinfected control group (Group A) matched the bacteria found on the formaldehyde disinfected egg, leading us to suspect a disinfection error. Hatching eggs treated with formaldehyde exhibited the characteristic formaldehyde smell after disinfection. Under secure conditions, formaldehyde shows in this trial a high disinfection efficacy and a good reproduction capacity of the results presented in the *in vitro* studies. Even though the disinfection efficacy of formaldehyde is high [27, 43], some other factors such as negative effect on workers due to its potential carcinogenic, toxic and mutagenic side effects [29, 44], has promoted the search of alternatives to this disinfection method [24, 29, 45–47]. The multiple negative side effects for the people working with this method, had created speculations regarding the possible prohibition of this method in the future, highlighting the need for alternatives [47, 48].

One of the most common disinfection alternatives to formaldehyde is a mixture of hydrogen peroxide + alcohol which was tested in this study. The low hazardous characteristics have made this method one of the preferred options for hatcheries to use instead of formaldehyde [49]. In our previous study [22], this method presented an efficient disinfection effect against ESBL producing *E*. *coli*, and other publications have reported a positive effect on gram-negative and positive bacteria such as *Pseudomonas fluorescens*, *Proteus sp*. *and Staphylococcus aureus* [24, 50]. Nevertheless this study showed a non-efficacious disinfection on field trials. The re-isolation rates presented using this method did not show a significant difference to the non-disinfected group, with an average of 8.7 x 10^3^ cfu/ml with a standard error of 8.2 x 10^2^ cfu/ml. The bacteria that were re-isolated and identified in this study showed the prevalence of gram-positive bacteria (i.e. *Staphylococcus lentus*) on the eggshell after disinfection. The same bacteria were found on the non-disinfected group, leading us to assume that the disinfection was not effective. Although the literature mentions the efficacy of this method on various bacteria [22, 50], the field trial conditions might have influenced the method´s efficacy. The product concentrations and method requirements were fulfilled, but the disinfection did not perform as expected based on previous *in vitro* trial results [22]. This can be due to the fact that in the *in vitro* trials only gram-negative bacteria were tested, while in the field trials, the efficacy was measured on the method’s ability to eliminate the complete microbiome on the eggshell surface, where mainly gram-positive bacteria was re-isolated. In this study, a mixture of hydrogen peroxide + alcohol was used for the disinfection which goes along with publications [50], explaining that the mixture of hydrogen peroxide with other ingredients such as alcohols or acids can improve the disinfection efficacy. An important fact to consider is that this method has no negative impact on hatchability or animal performance when compared to the gold standard formaldehyde, as demonstrated in parallel studies [34]. Although the disinfection efficacy was not as high as expected, the application proved to be suitable for larger egg numbers and easy to implement. The use of the same transportation trolleys and disinfection chambers allowed an easy and time-efficient process, and the adaptation of multiple nozzles in the disinfection room was possible without the need of new infrastructure. The product could be used without any risk of corrosion for the plastic and/or metal surfaces in the disinfection chamber.

Another method tested in the present investigation was peracetic acid in micro cages, which presented a significant difference to the non-disinfected group, but was not as effective as formaldehyde in terms of bacterial re-isolation rates. For this disinfection method, a difference in efficacy between the two flocks was observed as shown in Fig 1.

The disinfection process was carried out by an expert using a hig-pressure foaming gun in accordance with the manufacturer’s recommendations. Factors such as physical fatigue of the foaming gun operator could have played an important role on the re-isolation rates of the eggs. The density of eggs in the trolleys created “shadow” zones, and if the foaming gun operator missed one angle, the eggs in that area may not have been disinfected, leading to persistence of the eggshell microorganisms.

Regarding the suitability for large scale production, a new infrastructure capable of providing a non-human controlled disinfection could offer a more homogenous and secure disinfection compared to the one performed during this study. Another aspect to consider is the corrosion capacity of peracetic acid on metal and floor coating, as well as the intense smell of the product, which might require adaptations to the infrastructure.

Besides the potential negative effect of the acidity in the hatchery, multiple studies show that the “wetting” or washing of the eggs before incubation present during this method could lead to a higher bacterial colonization of the egg [51, 52].

In an effort to explore innovative techniques that could substitute formaldehyde in the future, a parallel test was performed using low energy electron beam disinfection. However, the prototype machine was not suitable for large scale production, therefore, the trial was compared only against the non-disinfected group and the reference group (formaldehyde) which was performed in parallel.

The bacterial disinfection capacity of the method is promising, with a re-isolation rate of 0.1 cfu/ml with a standard error of 0.166 cfu/ml reaching a disinfection efficacy that was not significantly different to the formaldehyde gold standard group. However, it is important to note that the low electron beam disinfection was limited to a disinfection batch size of 16 eggs at-a-time, eliminating the possibility to evaluate the suitability for large scale production. To assess the disinfection capacity of this method, further studies must be performed to see how the adaptation of this prototype to a large scale will affect the disinfection capacity. Nevertheless, the results presented during this trial, as well as the lack of negative side effect post hatching [26, 53], suggest that this method has potential as an alternative to formaldehyde once the appropriate field infrastructure is available.

## Conclusion

This study showed that there are effective hatching egg bacterial disinfection methods that work under field conditions, but also identified some drawbacks. When reproducing the different disinfection methods under field conditions, multiple factors played an important role which were not present during the *in vitro* testing. Infrastructure, corrosion risk, human application error, time, and efficiency should be considered when selecting and developing a new disinfection method. The disinfection capacity of the three alternative methods was strongly affected by the mode of application and must be regarded in context of the infrastructure of the method itself. Therefore, further studies with adapted methods and/or machines should be performed to further improve efficacious alternative methods for hatching egg disinfection suitable for large scale production. Additionally, further studies should be conducted to investigate the potential fungicidal effects of these methods.

## Supporting information

S1 Fig Graphical representation of experimental design for the evaluation of various methods for hatching egg disinfection efficacy in commercial broiler hatchery. (EPS)

S2 Fig Re-isolation rates of bacteria found on the eggshell with eggshell rinse method from repetition trial of control and disinfection groups. (EPS)
